# Survival Analysis and Prediction Model for Pulmonary Sarcomatoid Carcinoma Based on SEER Database

**DOI:** 10.3389/fonc.2021.630885

**Published:** 2021-05-31

**Authors:** Mingjing Chen, Qiao Yang, Zihan Xu, Bangyu Luo, Feng Li, Yongxin Yu, Jianguo Sun

**Affiliations:** ^1^ Cancer Institute, Xinqiao Hospital, Army Medical University, Chongqing, China; ^2^ Chongqing Key Laboratory of Infectious Diseases and Parasitic Diseases, Department of Infectious Diseases, The First Affiliated Hospital of Chongqing Medical University, Chongqing, China; ^3^ Department of Ultrasound, The 941^st^ Hospital of the PLA Joint Logistic Support Force, Xining, China; ^4^ Lung Cancer Center, Cancer Center and State Key Laboratory of Biotherapy, West China Hospital of Sichuan University, Chengdu, China

**Keywords:** clinical characteristics, prediction model, nomogram, pulmonary sarcomatoid carcinoma, SEER database

## Abstract

**Objective:**

This study aimed to investigate the incidence of the pulmonary sarcomatoid carcinoma (PSC), to compare the clinical characteristics and overall survival (OS) of patients with PSC and those with other non-small-cell lung cancer (oNSCLC), so as to analyze the factors affecting the OS of patients with PSC and construct a nomogram prediction model.

**Methods:**

Data of patients with PSC and those with oNSCLC diagnosed between 2004 and 2015 from the Surveillance, Epidemiology, and End Results database were collected. The age-adjusted incidence of PSC was calculated. The characteristics of patients with PSC and those with oNSCLC were compared, then the patients were matched 1:2 for further survival analysis. Patients with PSC were randomly divided into training set and testing set with a ratio of 7:3. The Cox proportional hazards model was used to identify the covariates associated with the OS. Significant covariates were used to construct the nomogram, and the C-index was calculated to measure the discrimination ability. The accuracy of the nomogram was compared with the tumor–node–metastasis (TNM) clinical stage, and the corresponding area under the curve was achieved.

**Results:**

A total of 1049 patients with PSC were enrolled, the incidence of PSC was slowly decreased from 0.120/100,000 in 2004 to 0.092/100,000 in 2015. Before PSM, 793 PSC patients and 191356 oNSCLC patients were identified, the proportion of male, younger patients (<65 years), grade IV, TNM clinical stage IV was higher in the PSC. The patients with PSC had significantly poorer OS compared with those with oNSCLC. After PSM, PSC still had an extremely inferior prognosis. Age, sex, TNM clinical stage, chemotherapy, radiotherapy, and surgery were independent factors for OS. Next, a nomogram was established based on these factors, and the C-indexs were 0.775 and 0.790 for the training and testing set, respectively. Moreover, the nomogram model indicated a more comprehensive and accurate prediction than the TNM clinical stage.

**Conclusions:**

The incidence of PSC was slowly decreased. PSC had a significantly poor prognosis compared with oNSCLC. The nomogram constructed in this study accurately predicted the prognosis of PSC, performed better than the TNM clinical stage.

## Introduction

Pulmonary sarcomatoid carcinoma (PSC) is a rare subtype of non-small-cell lung cancer (NSCLC), accounting for only 0.1%–0.4% of lung cancers (1). PSC refers to poorly differentiated NSCLC containing sarcoma or sarcoma-like components or carcinomas consisting of spindle cells and giant cells (2). According to the 2015 World Health Organization (WHO) classification of lung tumors and International Classification of Disease for Oncology, 3^rd^ Edition (ICD-O-3), PSC was classified into five categories: pleomorphic carcinoma, spindle cell carcinoma, giant cell carcinoma, carcinosarcoma, and pulmonary blastoma (3). Previous studies showed that PSC was more aggressive than other non-small-cell lung cancer (oNSCLC) and had a worse prognosis (4–6).

At present, the increasing number of studies on PSC are case reports or retrospective analyses focusing on the clinical-pathological characteristics and prognostic factors (6, 7). Despite numerous efforts to study the features of PSC, large population-based study never specifically investigated the incidence of PSC. The Surveillance, Epidemiology, and End Results (SEER) database is a systematic population-based cancer database. Therefore, this study was conducted to provide an overview of PSC incidence based on the data of SEER database.

Besides, only a few studies provided limited information about the detailed distinction between PSC and oNSCLC, and constructed a prediction model for PSC without external validation (8). Hence, this study also aimed to compare the clinicopathological characteristics and survival outcomes with oNSCLC, to explore the clinical features related to PSC overall survival (OS), and to construct and validate a nomogram prediction model.

## Methods

### Data Source

The patient data were obtained from the SEER 18 registries (www.seer.cancer.gov) of the National Cancer Institute using the SEER*Stat software (SEER*Stat 8.3.6). The database (1975–2016) covered about 27.8% of the American population and was released in April 2019 based on the November 2018 submission.

The inclusion criteria were as follows: patients with site record “lung and bronchus” and “one primary only”, diagnosed from 2004 to 2015. The diagnosis was confirmed by positive histology, and the type of reporting source was not autopsy or death certificate. Collectively, pleomorphic carcinoma (8022/3), giant cell carcinoma (8031/3), spindle cell carcinoma (8032/3), pulmonary blastoma (8972/3), and carcinosarcoma (8980/3) were grouped under the term PSC, and adenocarcinoma (8140/3, 8144/3, 8230/3, 8250/3, 8253/3, 8254/3, 8260/3, 8333/3, 8480/3, 8551/3), squamous cell carcinoma (8070/3–8072/3, 8083/3), adenosquamous carcinoma (8560/3), large cell carcinoma (8012/3), and others (8200/3, 8240/3, 8249/3, 8430/3, 8562/3) were grouped under the term oNSCLC. The patients lacking data about tumor, node, and metastases (TNM) clinical stage (American Joint Committee on Cancer Staging Manual, Sixth Edition) were excluded.

### Study Design

**Figure 1:**
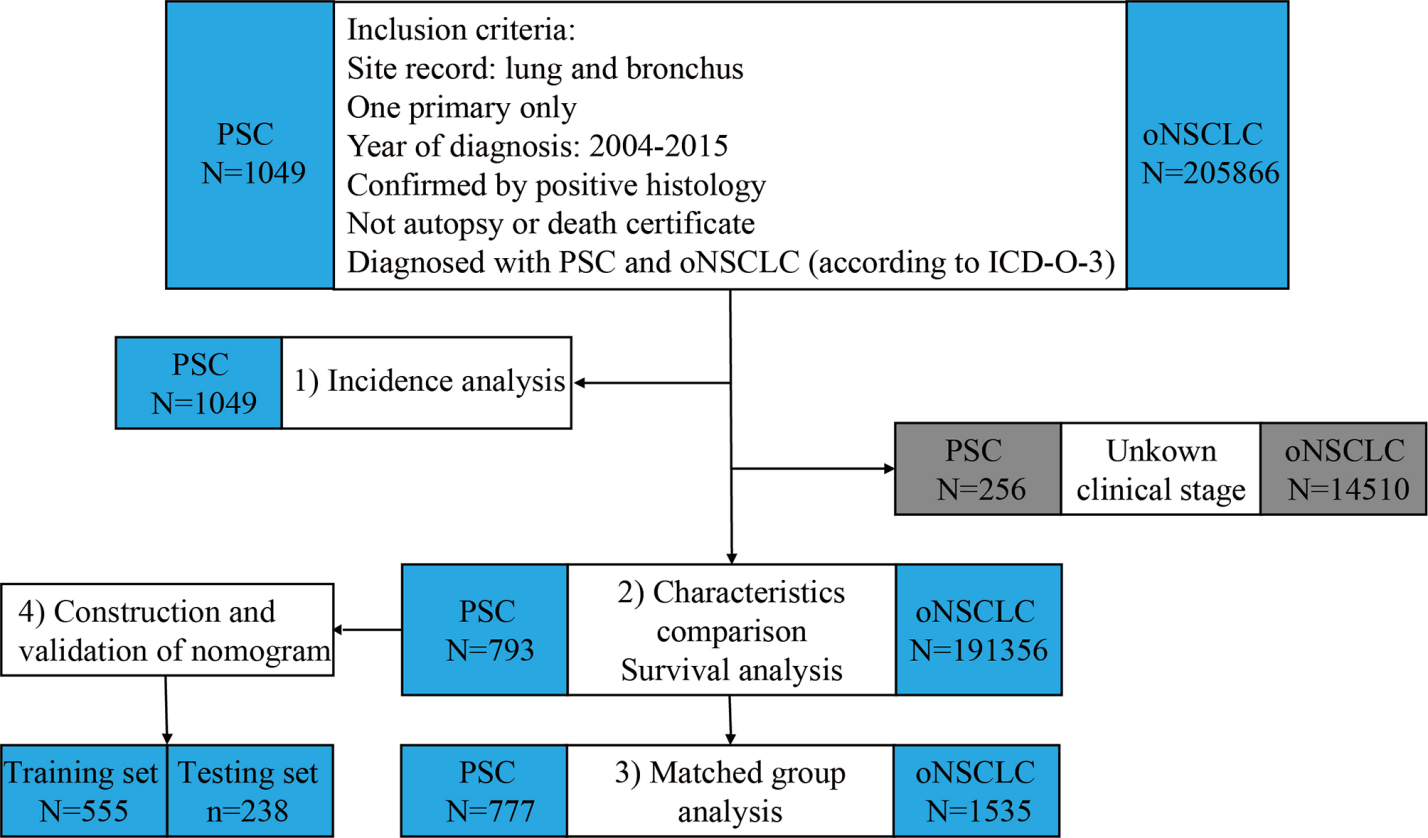
Flowchart of study design and patient selection. PSC, pulmonary sarcomatoid carcinoma; oNSCLC, other non-small-cell lung cancer.

The process and study design are presented in a flow-chart (**Figure 1**). The age-adjusted incidence of PSC was calculated with the patients with PSC. Then, the patients with PSC and the patients with oNSCLC were matched 1:2 between for further survival analysis using propensity score matching (PSM). Eligible patients with PSC were randomly divided into training set and testing set with a ratio of 7:3, and prognostic nomogram to predict 1-year survival for PSC was constructed based on training set and was validated using concordance index (C-index) and calibration curves in two sets. The total nomogram score of each patient was obtained, and the corresponding area under the curve (AUC) was achieved to compare the accuracy of the nomogram with the TNM clinical stage.

### Covariates

The study covariates included age at diagnosis, sex, race, year of diagnosis, grade, laterality, TNM clinical stage, chemotherapy, radiotherapy, surgery, survival months, and vital status.

### Statistical Analysis

Covariates were presented as frequency and percentages and compared using Pearson’s chi-square test. The age-adjusted incidence was calculated with Rate Session in the SEER*STAT software. PSM method was used to balance baseline covariates between PSC and oNSCLC. Survival analysis was accomplished by the Kaplan–Meier method and the log-rank test. In the PSC group, the Cox proportional hazards model was used to identify the covariates associated with OS and calculate the hazard ratio (HR) with 95% confidence interval (CI). The results were displayed using the forest plot. Based on the Cox model, significant covariates (*P* < 0.05) were used to construct the nomogram and the C-index was calculated to measure the discrimination ability. Statistical analyses were performed using the IBM SPSS Statistics, version 23.0, and the “survival,” “MatchIt,” “createtableone,” “love.plot,” “rms,” “nomogramEx,” “nomogramFormula,” and “survivalROC” packages in the R version 4.0.0 (http://www.r-project.org/).

## Results

### Annual Incidence

**Figure 2:**
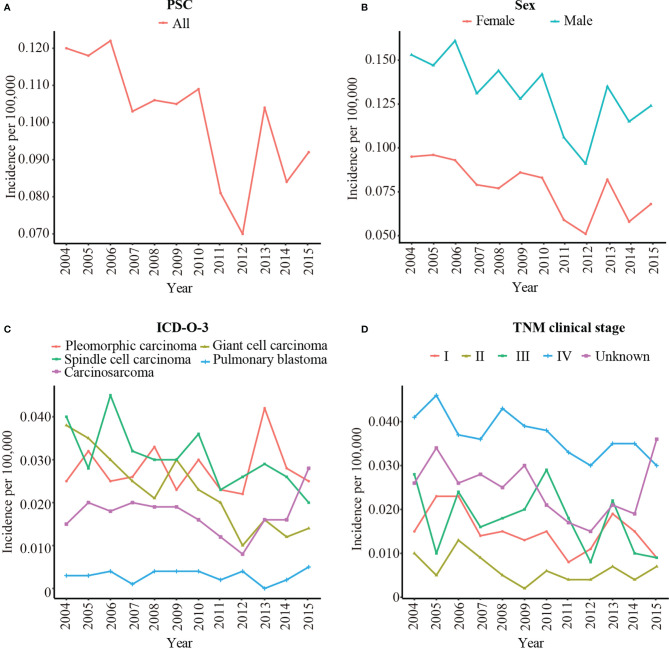
The age-adjusted incidence of PSC from 2004 to 2015 by sex, pathological type, and TNM clinical stage. **(A)** The age-adjusted incidence of all patients with PSC; **(B)** The age-adjusted incidence of PSC by sex; **(C)** The age-adjusted incidence of PSC by pathological type; **(D)** The age-adjusted incidence of PSC by TNM clinical stage. PSC, pulmonary sarcomatoid carcinoma; TNM clinical stage, tumor–node–metastasis clinical stage; ICD-O-3, International Classification of Disease for Oncology, 3^rd^ Edition.

A total of 1049 patients with PSC were enrolled to analysis the rate of incidence. The age-adjusted incidence of PSC was calculated based on the SEER 18 registries. Overall, the incidence of PSC was slowly decreased from 0.120/100,000 in 2004 to 0.092/100,000 in 2015 (**Figure 2A**). The incidence of male was obviously higher than female (**Figure 2B**). Among pathological subgroups, the incidence of carcinosarcoma increased from 0.015/100,000 in 2004 to 0.028/100,000 in 2015, and the incidence of giant cell carcinoma and spindle cell carcinoma decreased from 0.038/100,000 in 2004 to 0.014/100,000 in 2015 and 0.040/100,000 in 2004 to 0.020/100,000 in 2015, respectively. The incidence of pulmonary blastoma was stable (**Figure 2C**). In addition, the incidence decreased significantly in TNM clinical stage III from 0.028/100,000 in 2004 to 0.009/100,000 in 2015 (**Figure 2D**). **Supplementary Table 1** shows the detailed incidence data of PSC.

### Patient Characteristics

Of the 1049 patients with PSC and 205866 patients with oNSCLC in the primary SEER database, patients who had unknown TNM clinical stage (PSC: *N* = 256; oNSCLC: *N* = 14510) were excluded. Thus, 793 patients with PSC and 191356 patients with oNSCLC were identified (**Figure 1**).

The clinicopathological characteristics between two groups were shown as follows. A majority were men, in both PSC (59.4%) and oNSCLC (54.1%, *P* = 0.003), and most of them were white (80.3% *vs* 80.0%, *P* = 0.218). In total, 58.3% of the patients with PSC versus 62.2% of the patients with oNSCLC were older than 65 years (*P* = 0.023). PSCs had a significantly lower proportion during 2010–2015 (48.0% *vs* 55.5%, *P* < 0.001). Compared with oNSCLC, significantly more patients with PSC had grade IV tumors (14.9% *vs* 1.5%, *P* < 0.001) as well as TNM clinical stage IV (49.1% *vs* 45.9%, *P* < 0.001). More patients with oNSCLC received chemotherapy (45.8% *vs* 40.6%, *P* = 0.004), and radiotherapy (41.5% *vs* 36.6%, *P* = 0.005). More patients with PSC underwent surgery (36.9% *vs* 28.9%, *P* < 0.001). No significant difference was observed in laterality (**Table 1**).

**Table 1:** Comparison of clinicopathological characteristics between PSC and oNSCLC.

Characteristic	Before PSM			After PSM		
	PSC, *n* = 793, (%)	oNSCLC, *n* = 191356, (%)	*P*	PSC, *n* = 777, (%)	oNSCLC, *n* = 1535, (%)	*P*
**Age (year)**						
<65	331 (41.7)	72255 (37.8)	0.023	321 (41.3)	631 (41.1)	0.960
≥65	462 (58.3)	119101 (62.2)		456 (58.7)	904 (58.9)	
**Sex**						
Male	471 (59.4)	103539 (54.1)	0.003	460 (59.2)	909 (59.2)	1.000
Female	322 (40.6)	87817 (45.9)		317 (40.8)	626 (40.8)	
**Race**						
White	637 (80.3)	153151 (80.0)	0.218	632 (81.3)	1254 (81.7)	0.970
Black	105 (13.2)	23099 (12.1)		101 (13.0)	194 (12.6)	
Others	51 (6.4)	15106 (7.9)		44 (5.7)	87 (5.7)	
**Year of diagnosis**						
2004–2009	412 (52.0)	85096 (44.5)	<0.001	406 (52.3)	804 (52.4)	0.990
2010–2015	381 (48.0)	106260 (55.5)		371 (47.7)	731 (47.6)	
**Grade**						
I–II	6 (0.8)	58496 (30.6)	<0.001	6 (0.8)	12 (0.8)	0.961
III	309 (39.0)	59487 (31.1)		307 (39.5)	614 (40.0)	
IV	118 (14.9)	2929 (1.5)		108 (13.9)	201 (13.1)	
Unknown	360 (45.4)	70444 (36.8)		356 (45.8)	708 (46.1)	
**Laterality**						
Right	428 (54.0)	108271 (56.6)	0.299	423 (54.4)	833 (54.3)	0.996
Left	335 (42.2)	75683 (39.6)		325 (41.8)	645 (42.0)	
Others	30 (3.8)	7402 (3.9)		29 (3.7)	57 (3.7)	
**TNM stage**						
I	153 (19.3)	44825 (23.4)	<0.001	148 (19.0)	288 (18.8)	0.997
II	66 (8.3)	10790 (5.6)		64 (8.2)	124 (8.1)	
III	185 (23.3)	47863 (25.0)		182 (23.4)	361 (23.5)	
IV	389 (49.1)	87878 (45.9)		383 (49.3)	762 (49.6)	
**Chemotherapy**						
Yes	322 (40.6)	87565 (45.8)	0.004	311 (40.0)	612 (39.9)	0.978
No/Unknown	471 (59.4)	103791 (54.2)		466 (60.0)	923 (60.1)	
**Radiotherapy**						
Yes	290 (36.6)	79500 (41.5)	0.005	283 (36.4)	557 (36.3)	0.985
No/Unknown	503 (63.4)	111856 (58.5)		494 (63.6)	978 (63.7)	
**Surgery**						
Yes	293 (36.9)	55359 (28.9)	<0.001	282 (36.3)	548 (35.7)	0.814
No/Unknown	500 (63.1)	135997 (71.1)		495 (63.7)	987 (64.3)	

PSM, propensity score matching; PSC, pulmonary sarcomatoid carcinoma; oNSCLC, other non-small-cell lung cancer; TNM stage, tumor–node–metastasis stage.

### PSM for PSC and oNSCLC

Thus, the PSM method was used to balance all characteristics, including age, sex, race, year of diagnosis, grade, laterality, TNM clinical stage, chemotherapy, radiotherapy, and surgery between the two groups.

**Figure 3:**
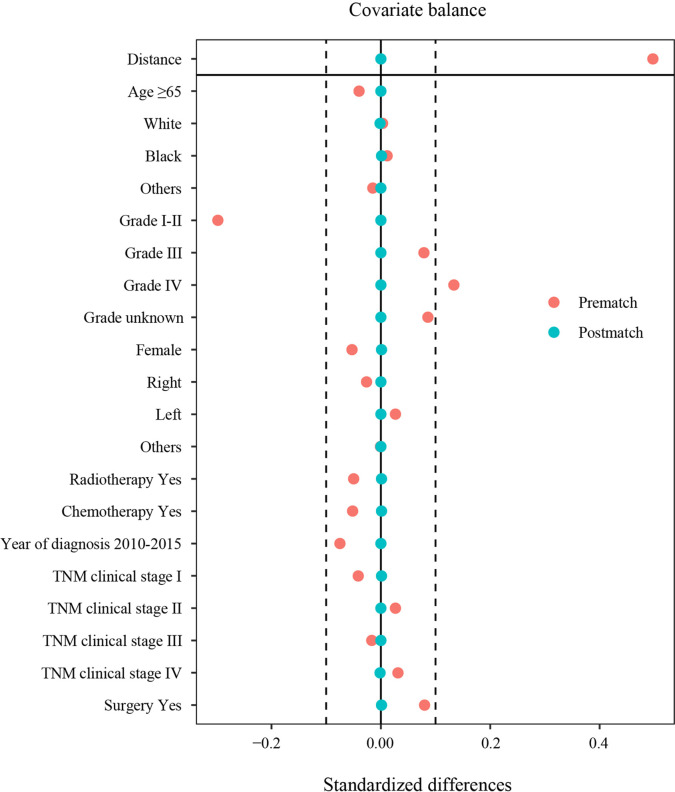
Standardized differences in characteristics before and after PSM. TNM clinical stage, tumor–node–metastasis clinical stage; PSM, propensity score matching.

After PSM, the clinicopathological characteristics between 777 patients with PSC and 1535 patients with oNSCLC were shown as follows. Most of the patients were men, in both PSC (59.2%) and oNSCLC (59.2%, *P* = 1.000). Approximately 59% patients were older than 65 years in two groups (*P* = 0.960). Grade IV tumors (13.9% *vs* 13.1%, *P* = 0.961) as well as TNM clinical stage IV (49.3% *vs* 49.6%, *P* = 0.997) were balanced in two groups, and other covariates, including race, year of diagnosis, laterality, chemotherapy, radiotherapy, and surgery also showed no significantly difference (**Table 1** and **Figure 3**).

### Survival Analysis of PSC and oNSCLC

**Figure 4:**
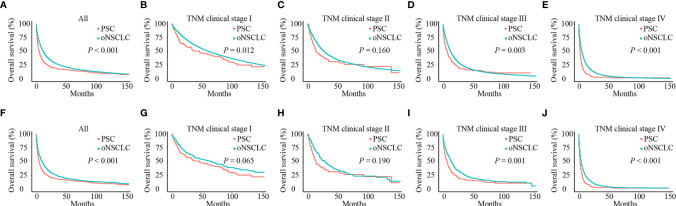
Survival curves of patients with PSC and oNSCLC. **(A)** Before PSM, OS in patients with PSC and oNSCLC; **(B–E)** Before PSM, OS in patients with PSC and oNSCLC at different clinical stage; **(F)** After PSM, OS in patients with PSC and oNSCLC; **(G–J)** After PSM, OS in patients with PSC and oNSCLC at different clinical stage. PSC, pulmonary sarcomatoid carcinoma; oNSCLC, other non-small-cell lung cancer; PSM, propensity score matching.

Before PSM, patients with PSC had significantly poorer OS compared with patients with oNSCLC (mOS: 5.0 months *vs* 12.0 months, *P* < 0.001) (**Figure 4A**). Patients with PSC who had stage I (mOS: 46.0 months *vs* 61.0 months, *P* = 0.012), stage III (mOS: 7.0 months *vs* 12.0 months, *P* = 0.003) and stage IV (mOS: 2.0 months *vs* 5.0 months, *P* < 0.001) revealed an inferior survival compared with oNSCLC, respectively. But stage II showed no difference (mOS: 14.0 months *vs* 27.0 months, *P* = 0.160; **Figures 4B–E**).

After adjusting for covariates (age, sex, year of diagnosis, grade, TNM clinical stage, chemotherapy, radiotherapy and surgery), PSC still performed worse survival (mOS: 5.0 months *vs* 10.0 months, *P* < 0.001; **Figure 4F**). Also, patients with PSC revealed significantly inferior survival compared with patients with oNSCLC at stage III (mOS: 6.0 months vs 14.0 months, *P* = 0.001) and stage IV (mOS: 2.0 months *vs* 4.0 months, *P* < 0.001; **Figures 4I, J**), respectively. But patients with PSC and patients with oNSCLC had stage I (mOS: 46.0 months *vs* 65.0 months, *P* = 0.065) and stage II (mOS: 14.0 months *vs* 29.0 months, *P* = 0.190) showed no difference, respectively (**Figures 4G, H**).

### Multivariate Analysis of PSC

A total of 793 patients were included in the PSC group. With a ratio of 7:3, patients with PSC were randomly assigned to the training set (N=555) and testing set (N=238). **Supplementary Table 2** lists the baseline characteristics, no significant difference was found between the two sets.

**Figure 5:**
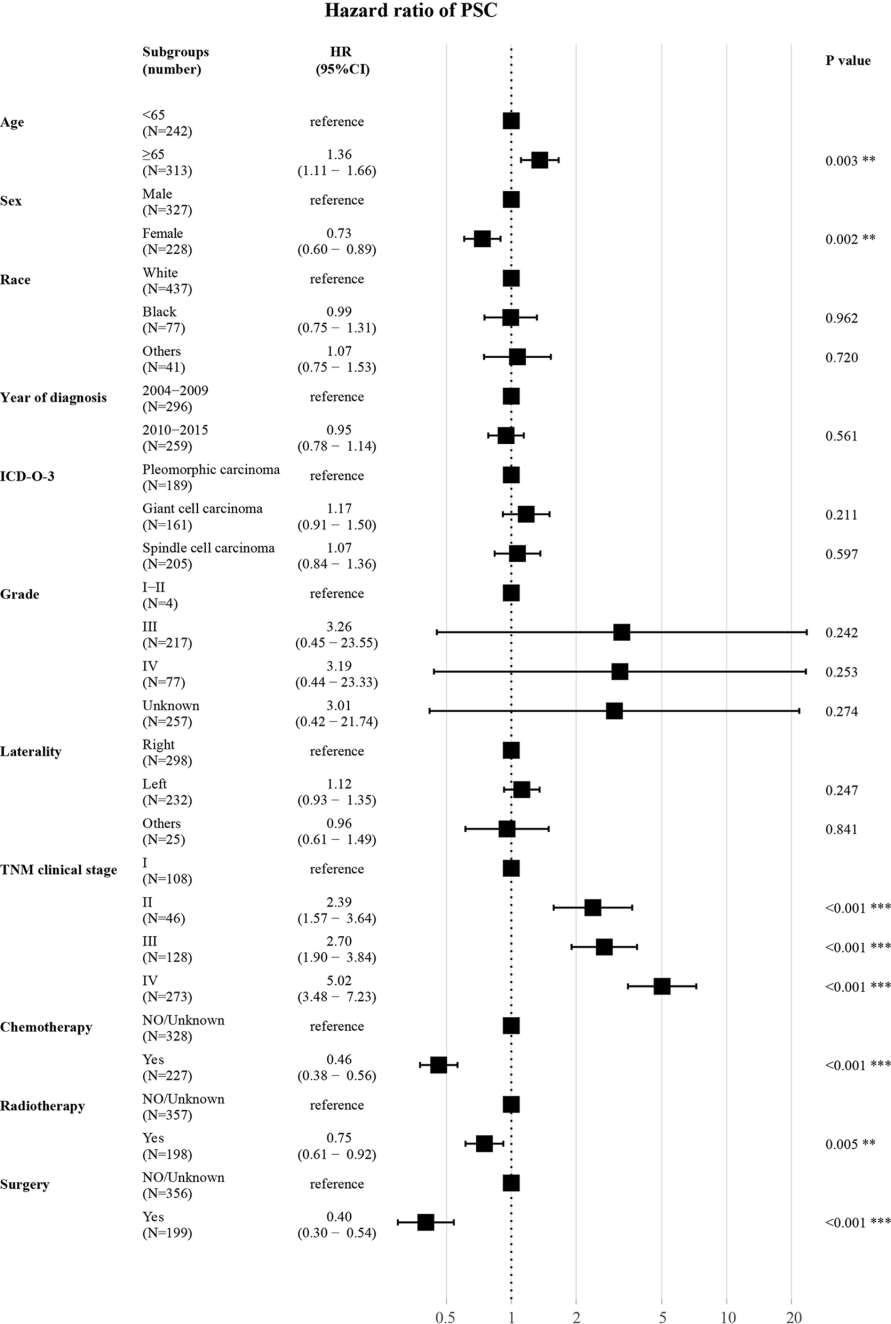
Forest plot for multivariate analysis of the effect of different variables. HR, hazard ratio; CI, confidence interval; ICD-O-3, International Classification of Disease for Oncology, 3^rd^ Edition; TNM clinical stage, tumor–node–metastasis clinical stage. **P < 0.01; ***P < 0.001.

As shown (**Figure 5**), in the training set, age (≥65 years, HR 1.36, *P* = 0.003) had a correlation with worse prognosis while female (HR 0.73, *P* = 0.002) was associated with better OS. The TNM clinical stage (II *vs* I, HR 2.39, *P* < 0.001; III *vs* I, HR 2.70, *P* < 0.001; IV *vs* I, HR 5.02, *P* < 0.001) were also covariates having effect on OS. Patients who received chemotherapy (HR 0.46, *P* < 0.001) or radiotherapy (HR 0.75, *P* = 0.005) or underwent surgery (HR 0.40, *P* < 0.001) experienced superior survival compared with those who did not. Other characteristics (race, year of diagnosis, pathological type, grade, and laterality) had no statistically significant difference (*P* > 0.05) in the model.

### Construction and Validation of the Nomogram

**Figure 6:**
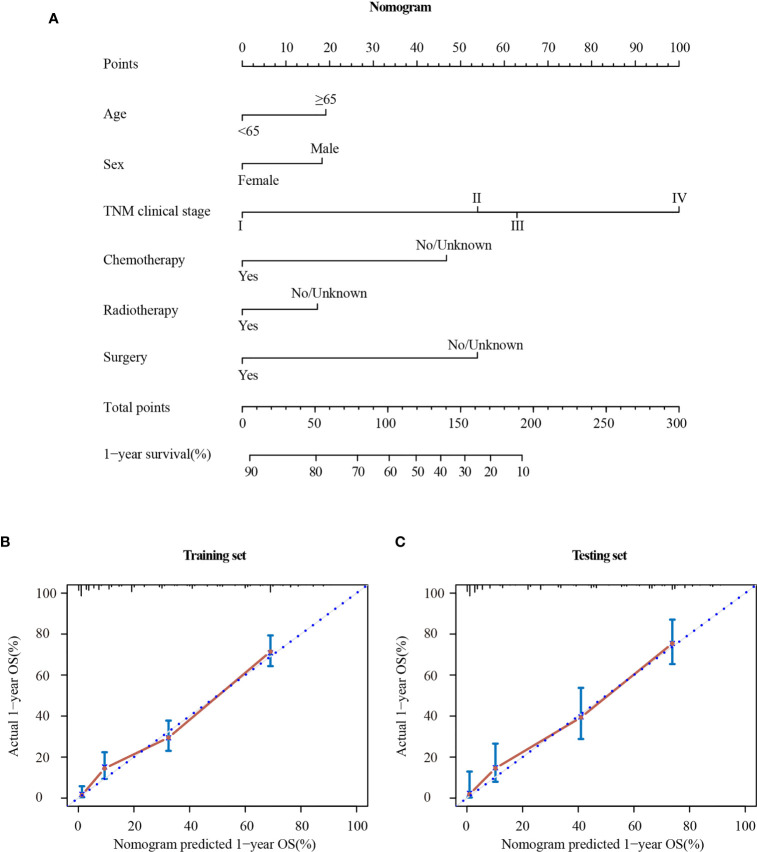
Nomogram **(A)** and calibration curve in the training set **(B)** and testing set **(C)**. TNM clinical stage, tumor–node–metastasis clinical stage.

The Cox regression analysis demonstrated that age, sex, TNM clinical stage, chemotherapy, radiotherapy, and surgery were independent prognostic factors for OS. So a nomogram was established to predict 1-year survival based on the results (**Figure 6A**). The TNM clinical stage was the largest contributing covariate to prognosis, followed by surgery and chemotherapy. In addition, age, sex and radiotherapy also presented an impact on OS. Each subtype of significant characteristics corresponded to a unique point. The total points of every patient were calculated. It was convenient to estimate the probability of 1-year survival by locating it on the scale. **Supplementary Table 3** shows the detailed score for each characteristic.

The stability of the nomogram was validated using the calibration plot in the training set (**Figure 6B**) and testing set (**Figure 6C**), respectively. The calibration curves displayed high internal and external consistency with the actual observation for 1-year survival, and achieving C-indexs of 0.775 and 0.790 for the training and testing set, respectively.

### Comparison of the Predictive Accuracy for OS Between Nomogram and TNM Clinical Stage

**Figure 7:**
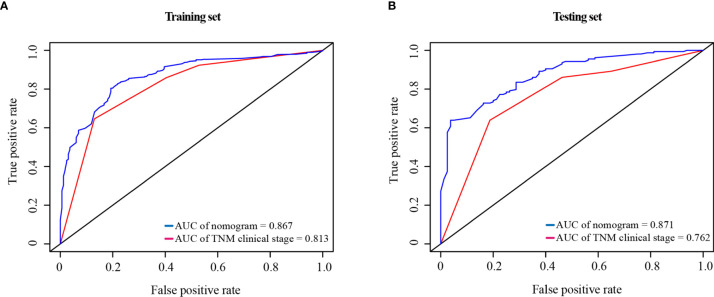
ROC curve of the nomogram and the TNM clinical stage in the training set **(A)** and testing set **(B)**. AUC, area under the curve; ROC, receiver operating characteristic; TNM clinical stage, tumor–node–metastasis clinical stage.

Compared with the TNM clinical stage, in the training test, the AUCs of nomogram and TNM clinical stage were 0.867 and 0.813, respectively; and in the testing set, the AUCs were 0.871 and 0.762, respectively. As shown in **Figure 7**, the nomogram had better prediction accuracy for the 1-year survival probability compared to TNM clinical stage.

## Discussion

This large retrospective study showed that PSC was a rare cancer, accounting for less than 0.5% of oNSCLC, as described in previous studies (4, 5). It is remarkable that the incidence of PSC was slowly decreased. As for pathological, the incidence of carcinosarcoma was significant increased. PSC occurred more frequently in the elder (≥65 years) and male individuals (9). In the current study, PSC had a highly aggressive behavior, with a significantly higher proportion of poorly differentiated tumors (grade IV: 14.9% *vs* 1.5%, *P <*0.001) compared with oNSCLC, and up to 49.1% of patients were in stage IV when diagnosed.

PSC is difficult to diagnose, leading to a poor prognosis (1, 4). In small-sample studies on PSC, mOS was 5–8.5 months (10–14). In previous SEER-based studies, mOS was 6.0 months for patients with all stages and 3.0 months for patients with advanced disease (4, 6). In this study, mOS was 5.0 months for PSC and 12.0 months for oNSCLC (*P* < 0.001) before PSM. After equalizing significantly different characteristics in PSC and oNSCLC, PSCs were still found to have a significantly poorer clinical outcome compared with oNSCLC (mOS: 5.0 m *vs* 10.0 m, *P* < 0.001).

The multivariate Cox proportional hazards model revealed that elderly, male patients with advanced clinical stage had a worse prognosis (2, 15), whereas receiving chemotherapy, radiotherapy, or surgery could prolong OS. At present, the standard treatment of PSC is controversial (5, 9). Surgery in the early-stage PSC has been demonstrated to provide optimal OS benefit, but a high risk of recurrence and adjuvant chemotherapy should be considered (2, 5, 6, 15). Previous studies indicated that adjuvant chemotherapy after surgery was effective in improving survival outcomes (15, 16), and large population-based studies also revealed the benefit of chemotherapy (7). On the contrary, in a study on 69 patients with PSC, Liang et al. reported that adjuvant chemotherapy could not improve OS (5). PSC was a chemorefractory cancer in previous studies (9, 11, 17). Patients with PSC who received first-line platinum-based chemotherapy did not experience significant benefits (mOS: 7.0 months *vs* 5.3 months, *P* = 0.096) (18). Few studies on the effect of radiotherapy on the prognosis of PSC and a small-sample study reported that patients who received radiotherapy had a worse mOS (5.0 m *vs* 6.0 m, *P* < 0.001) (6, 19). However, a retrospective study based on SEER showed that radiotherapy improved the survival in stage I-III patients with PSC (8). In this multivariate analysis, both chemotherapy (HR 0.46, *P* < 0.001) and radiotherapy (HR 0.75, *P* = 0.005) were protective factors and improved the survival. By far, data showing that chemotherapy or radiotherapy prolongs survival is insufficient, and requires further prospective research.

In recent studies, MET exon 14 skipping mutations were found to be enriched in PSC, and the incidence rate was as high as 31.8% (20), which could be a potential target for therapy using MET inhibitors, such as volitinib, capmatinib, and tepotinib (21–23). In addition, patients with PSC had an increased tendency for the high expression of tumor mutation burden (>20 mutations per Mb) compared with those with oNSCLC (20% *vs* 14%, *P* = 0.056) (22). At the same time, PSC also had a high expression of PD-L1, suggesting that targeting PD-1/PD-L1 might be a potential treatment regimen for PSC. Patients with PSC who received PD-1/PD-L1 inhibitors exhibited high response rates and prolonged overall survival (24–31). Also some novel biomarkers were investigated to predict the survival outcome of PSC, such as CD8+ tumor-infiltrating lymphocytes, the epithelial−mesenchymal transition transcription factors (Twist1), the change of neutrophil-to-lymphocyte ratio, KRAS mutations and c-MET overexpression (32–36).

In this study, six features, including age, sex, TNM clinical stage, chemotherapy, radiotherapy, and surgery, were used to construct a nomogram prognostic model. The total score was calculated using the quantitative score of each feature, and the 1-year survival rate was predicted scientifically and accurately. The C-indexs of this model were 0.775 and 0.790 in internal and external validation, respectively, indicating good agreement between predicted and actual 1-year survival. Next, the nomogram model was compared with the conventional TNM clinical stage and found to be superior in the 1-year survival of AUC both in the training set and testing set, indicating a more comprehensive and accurate prediction. This model could be used to individualize prognostic assessment and might serve an effective diagnostic tool for making treatment-related decisions (37, 38).

This study had several limitations. First, the variables enrolled were restricted; some important characteristics related to prognosis were not included in this study, such as smoking status, performance status score, gene mutation detection by next generation sequencing, MET expression detection, and PD-L1 immunohistochemistry assay. Second, treatment information was limited, without target therapy, immunotherapy, etc. The database only contained the status of surgery, chemotherapy, and radiotherapy, but some of which were not known. Third, the construction of this model was based on retrospective data and requires further confirmation.

In conclusion, the incidence of PSC was slowly decreased, and for pathological subgroups, the incidence of carcinosarcoma was increased. PSC had a significantly poor prognosis compared with oNSCLC. The nomogram constructed in this study accurately predicted the prognosis of PSC, and performed better than the TNM clinical stage. This model is expected to help pathologist and oncologist in designing clinical strategies.

## Data Availability Statement

Publicly available datasets were analyzed in this study. This data can be found here: The SEER 18 registries (www.seer.cancer.gov) of the National Cancer Institute using the SEER*Stat software (SEER*Stat 8.3.6).

## Author Contributions

JS, MC, and QY designed the study. MC, QY, and ZX contributed to data selection and assembly. BL, FL, and YY analysed data. MC and QY were involved in drafting the manuscript. JS critically revised the manuscript. All authors contributed to the article and approved the submitted version.

## Funding

The study was supported by the National Natural Science Foundation of China (81773245, 81972858), the Clinical Research Project of Army Medical University (2018XLC1010), and the Chongqing Science and Technology Leading Talents Program (cstccxljrc201910).

## Conflict of Interest

The authors declare that the research was conducted in the absence of any commercial or financial relationships that could be construed as a potential conflict of interest.
