# Supplementary material for: Survival Analysis and Prediction Model for Pulmonary Sarcomatoid Carcinoma Based on SEER Database
Source: Front Oncol. 2021 May 31;11:630885. doi: 10.3389/fonc.2021.630885 (PMC8201495; doi:10.3389/fonc.2021.630885)
Supplement: Supplementary file 1 [file DataSheet_1.zip › Sup Table 1.DOCX]

Supplementary Table 1. Incidence of pulmonary sarcomatoid carcinoma from 2004 to 2015.

| **Group** | **Year** | | | | | | | | | | | |
| --- | --- | --- | --- | --- | --- | --- | --- | --- | --- | --- | --- | --- |
|  | 2004 | 2005 | 2006 | 2007 | 2008 | 2009 | 2010 | 2011 | 2012 | 2013 | 2014 | 2015 |
| **All** | 0.120 | 0.118 | 0.122 | 0.103 | 0.106 | 0.105 | 0.109 | 0.081 | 0.070 | 0.104 | 0.084 | 0.092 |
| **Sex** |  |  |  |  |  |  |  |  |  |  |  |  |
| Male | 0.153 | 0.147 | 0.161 | 0.131 | 0.144 | 0.128 | 0.142 | 0.106 | 0.091 | 0.135 | 0.115 | 0.124 |
| Female | 0.095 | 0.096 | 0.093 | 0.079 | 0.077 | 0.086 | 0.083 | 0.059 | 0.051 | 0.082 | 0.058 | 0.068 |
| **ICD-O-3** |  |  |  |  |  |  |  |  |  |  |  |  |
| Pleomorphic carcinoma | 0.025 | 0.032 | 0.025 | 0.026 | 0.033 | 0.023 | 0.030 | 0.023 | 0.022 | 0.042 | 0.028 | 0.025 |
| Giant cell carcinoma | 0.038 | 0.035 | 0.030 | 0.025 | 0.021 | 0.030 | 0.023 | 0.020 | 0.010 | 0.016 | 0.012 | 0.014 |
| Spindle cell carcinoma | 0.040 | 0.028 | 0.045 | 0.032 | 0.030 | 0.030 | 0.036 | 0.023 | 0.026 | 0.029 | 0.026 | 0.020 |
| Pulmonary blastoma | 0.003 | 0.003 | 0.004 | 0.001 | 0.004 | 0.004 | 0.004 | 0.002 | 0.004 | 0 | 0.002 | 0.005 |
| Carcinosarcoma | 0.015 | 0.020 | 0.018 | 0.020 | 0.019 | 0.019 | 0.016 | 0.012 | 0.008 | 0.016 | 0.016 | 0.028 |
| **TNM clinical stage** |  |  |  |  |  |  |  |  |  |  |  |  |
| Ⅰ | 0.015 | 0.023 | 0.023 | 0.014 | 0.015 | 0.013 | 0.015 | 0.008 | 0.011 | 0.019 | 0.015 | 0.009 |
| Ⅱ | 0.010 | 0.005 | 0.013 | 0.009 | 0.005 | 0.002 | 0.006 | 0.004 | 0.004 | 0.007 | 0.004 | 0.007 |
| Ⅲ | 0.028 | 0.010 | 0.024 | 0.016 | 0.018 | 0.020 | 0.029 | 0.018 | 0.008 | 0.022 | 0.010 | 0.009 |
| Ⅳ | 0.041 | 0.046 | 0.037 | 0.036 | 0.043 | 0.039 | 0.038 | 0.033 | 0.030 | 0.035 | 0.035 | 0.030 |
| Unknown | 0.026 | 0.034 | 0.026 | 0.028 | 0.025 | 0.030 | 0.021 | 0.017 | 0.015 | 0.021 | 0.019 | 0.036 |

ICD-O-3, International Classification of Disease for Oncology, 3^rd^ Edition; TNM clinical stage, tumor–node–metastasis clinical stage.
