# Supplementary material for: Survival Analysis and Prediction Model for Pulmonary Sarcomatoid Carcinoma Based on SEER Database
Source: Front Oncol. 2021 May 31;11:630885. doi: 10.3389/fonc.2021.630885 (PMC8201495; doi:10.3389/fonc.2021.630885)
Supplement: Supplementary file 1 [file DataSheet_1.zip › Sup Table 2.DOCX]

Supplementary Table 2. Baseline characteristics in the training set and testing set.

| **Characteristic** | **Cohort** | | ***P*** |
| --- | --- | --- | --- |
|  | **Training set** | **Testing set** |  |
| **Age (year)** |  |  |  |
| <65 | 242 (43.6) | 89 (37.4) | 0.122 |
| ≥65 | 313 (56.4) | 149 (62.6) |  |
| **Sex** |  |  |  |
| Male | 327 (58.9) | 144 (60.5) | 0.736 |
| Female | 228 (41.1) | 94 (39.5) |  |
| **Race** |  |  |  |
| White | 437 (78.7) | 200 (84.0) | 0.152 |
| Black | 77 (13.9) | 28 (11.8) |  |
| Others | 41 (7.4) | 10 (4.2) |  |
| **Year of diagnosis** |  |  |  |
| 2004–2009 | 296 (53.3) | 116 (48.7) | 0.267 |
| 2010–2015 | 259 (46.7) | 122 (51.3) |  |
| **ICD-O-3** |  |  |  |
| Pleomorphic carcinoma | 189 (34.1) | 95 (39.9) | 0.262 |
| Giant cell carcinoma | 161 (29.0) | 66 (27.7) |  |
| Spindle cell carcinoma | 205 (36.9) | 77 (32.4) |  |
| **Grade** |  |  |  |
| Ⅰ–Ⅱ | 4 (0.7) | 2 (0.8) | 0.651 |
| Ⅲ | 217 (39.1) | 92 (38.7) |  |
| Ⅳ | 77 (13.9) | 41 (17.2) |  |
| Unknown | 257 (46.3) | 103 (43.3) |  |
| **Laterality** |  |  |  |
| Right | 298 (53.7) | 130 (54.6) | 0.265 |
| Left | 232 (41.8) | 103 (43.3) |  |
| Others | 25 (4.5) | 5 (2.1) |  |
| **TNM clinical stage** |  |  |  |
| Ⅰ | 108 (19.5) | 45 (18.9) | 0.993 |
| Ⅱ | 46 (8.3) | 20 (8.4) |  |
| Ⅲ | 128 (23.1) | 57 (23.9) |  |
| Ⅳ | 273 (49.2) | 116 (48.7) |  |
| **Chemotherapy** |  |  |  |
| Yes | 227 (40.9) | 95 (39.9) | 0.857 |
| No/Unknown | 328 (59.1) | 143 (60.1) |  |
| **Radiotherapy** |  |  |  |
| Yes | 198 (35.7) | 92 (38.7) | 0.473 |
| No/Unknown | 357 (64.3) | 146 (61.3) |  |
| **Surgery** |  |  |  |
| Yes | 199 (35.9) | 94 (39.5) | 0.372 |
| No/Unknown | 356 (64.1) | 144 (60.5) |  |

ICD-O-3, International Classification of Disease for Oncology, 3^rd^ Edition; TNM clinical stage, tumor–node–metastasis clinical stage.
