# Supplementary material for: Survival Analysis and Prediction Model for Pulmonary Sarcomatoid Carcinoma Based on SEER Database
Source: Front Oncol. 2021 May 31;11:630885. doi: 10.3389/fonc.2021.630885 (PMC8201495; doi:10.3389/fonc.2021.630885)
Supplement: Supplementary file 1 [file DataSheet_1.zip › Sup Table 3.DOCX]

Supplementary Table 3. Detailed nomogram score for every factor in the prognostic nomogram for PSC 1-year OS probability.

| Predictive factors |  |  |
| --- | --- | --- |
| Variable | Category | Score |
| Age (years) | < 65 | 0 |
|  | ≥ 65 | 19 |
| Sex | Male | 18 |
|  | Female | 0 |
| TNM clinical stage | Ⅰ | 0 |
|  | Ⅱ | 54 |
|  | Ⅲ | 63 |
|  | Ⅳ | 100 |
| Chemotherapy | Yes | 0 |
|  | No/Unknown | 47 |
| Radiation | Yes | 0 |
|  | No/Unknown | 17 |
| Surgery | Yes | 0 |
|  | No/Unknown | 54 |
| 1-year OS probability |  |  |
|  | Survival probability | Total score |
|  | 0.1 | 192 |
|  | 0.2 | 171 |
|  | 0.3 | 153 |
|  | 0.4 | 136 |
|  | 0.5 | 119 |
|  | 0.6 | 101 |
|  | 0.7 | 79 |
|  | 0.8 | 51 |
|  | 0.9 | 5 |

OS, overall survival.
